# A Conscious Resting State fMRI Study in SLE Patients Without Major Neuropsychiatric Manifestations

**DOI:** 10.3389/fpsyt.2018.00677

**Published:** 2018-12-07

**Authors:** Shuang Liu, Yuqi Cheng, Zhongqi Xie, Aiyun Lai, Zhaoping Lv, Yueyin Zhao, Xiufeng Xu, Chunrong Luo, Hongjun Yu, Baoci Shan, Lin Xu, Jian Xu

**Affiliations:** ^1^Department of Rheumatology and Immunology, First Affiliated Hospital of Kunming Medical University, Kunming, China; ^2^Yunnan Key Laboratory of Laboratory Medicine, Kunming, China; ^3^Department of Psychiatry, First Affiliated Hospital of Kunming Medical University, Kunming, China; ^4^Magnetic Resonance Imaging Center, The First Hospital of Kunming, Kunming, China; ^5^Key Laboratory of Nuclear Analysis, Institute of High Energy Physics, Chinese Academy of Sciences, Beijing, China; ^6^Key Laboratory of Animal Models and Human Disease Mechanisms, Kunming Institute of Zoology, Chinese Academy of Sciences, Kunming, China

**Keywords:** systemic lupus erythematosus patients without major neuropsychiatric manifestations (non-NPSLE patients), resting-state functional magnetic resonance imaging (rs-fMRI), regional homogeneity (ReHo), disease activity, anxiety, depression

## Abstract

Neuropsychiatric systemic lupus erythematosus (NPSLE) is one of the main causes of death in patients with systemic lupus erythematosus (SLE). Signs and symptoms of NPSLE are heterogeneous, and it is hard to diagnose, and treat NPSLE patients in the early stage. We conducted this study to explore the possible brain activity changes using resting state functional magnetic resonance imaging (rs-fMRI) in SLE patients without major neuropsychiatric manifestations (non-NPSLE patients). We also tried to investigate the possible associations among brain activity, disease activity, depression, and anxiety. In our study, 118 non-NPSLE patients and 81 healthy controls (HC) were recruited. Rs-fMRI data were used to calculate the regional homogeneity (ReHo) in all participants. We found decreased ReHo values in the fusiform gyrus and thalamus and increased ReHo values in the parahippocampal gyrus and uncus. The disease activity was positively correlated with ReHo values of the cerebellum and negatively correlated with values in the frontal gyrus. Several brain areas showed correlations with depressive and anxiety statuses. These results suggested that abnormal brain activities might occur before NPSLE and might be the foundation of anxiety and depression symptoms. Early detection and proper treatment of brain dysfunction might prevent the progression to NPSLE. More studies are needed to understand the complicated underlying mechanisms.

## Introduction

Systemic lupus erythematosus (SLE) is an autoimmune disease with multiorgan involvement and several typical autoantibodies. Central nervous system (CNS) involvement is common. Patients with neuropsychiatric signs and symptoms are classified as neuropsychiatric SLE (NPSLE) patients. NPSLE patients show poor prognosis, low quality of life and high mortality. In 1999, the American College of Rheumatology (ACR) defined 19 typical neuropsychiatric symptoms such as cerebrovascular disease (CVD), seizures, anxiety, and cognitive dysfunction (1). While obvious NPSLE symptoms such as CVD and seizures are well-recognized by doctors, subtle ones such as anxiety and mild cognitive dysfunction are underestimated. Early detection and interventions are very important for those patients. Clinical psychiatric evaluations, such as the Hamilton Anxiety Scale (HAMA) and the Hamilton Depression Scale (HAMD), are widely used in patients with anxiety and depressive disorders. Conventional magnetic resonance imaging (MRI) is used to evaluate structural abnormalities, and functional MRI (fMRI) is used to evaluate brain activity. Task-related fMRI and conscious resting state fMRI (rs-fMRI) are used to reveal different aspects of brain function.

In this study, we examined SLE patients without major neuropsychiatric manifestations or abnormal conventional MRI as non-NPSLE patients. We used conscious rs-fMRI to explore the possible changes of brain activity and tried to find the associations among brain activity, disease activity, and depression, and anxiety statuses.

## Materials and Methods

### Subjects

SLE patients in the inpatient and outpatient divisions of the Rheumatology and Immunology Department of First Affiliated Hospital of Kunming Medical University, a member unit of the Chinese SLE Treatment and Research Group (CSTAR), were recruited in this study from August of 2003 to August of 2015. All participants went through a standardized protocol and were evaluated by the same investigators throughout the course of the study. Before enrollment in the study, each participant provided written informed consent after receiving a complete description of the study. This research protocol was approved by the Institutional Review Board of Kunming Medical University, Yunnan Province, PR China (ClinicalTrials.gov: NCT00703742).

The inclusion criteria were as follows: (1) patients diagnosed as SLE according to the 1997 revised American College of Rheumatology (ACR) criteria for the classification of SLE (2) (2) patients between the ages of 15 and 60; and (3) patients or statutory guardians willing to attend this study and give written informed consent.

The exclusion criteria were as follows: (1) patients fitting the ACR diagnostic criteria for rheumatoid arthritis, systemic sclerosis, Sjögren's syndrome (primary or secondary) or other connective tissue diseases and drug-induced SLE; (2) patients with organic brain or neurological disorders that would disturb the structure or diffusion imaging of the brain (i.e., history of head trauma, Parkinson's disease or seizures); (3) patients with major active psychiatric manifestations, such as obvious disorganized behaviors and conscious disturbances; (4) patients with a history of substance abuse; (5) patients who were pregnant or suspected to be pregnant; (6) patients with contraindication to MRI, such as claustrophobia or cardiac pacemakers; (7) patients with serious clinical conditions that could cause cerebral atrophy, such as a history of arterial hypertension, diabetes mellitus, stroke or renal insufficiency; and (8) patients with structural abnormities of the brain identified by conventional T1- and T2-weighted MRI.

All of the recruited 118 SLE patients received the full sets of laboratory tests, disease activity evaluations, psychiatric evaluations, general questionnaires, and MRI scans. In addition, 81 healthy controls (HC) with age and gender matched were recruited. A rheumatologist and a neurologist performed the complete general physical examinations, including neurological examinations to all subjects to exclude major disorders. Psychiatric symptoms were screened by a psychiatrist using the Structured Clinical Interview for the Diagnostic and Statistical Manual of Mental Disorders (DSM)-IV Non-Patient Version (SCID-NP). All participants were right-handed Chinese Han people.

### Clinical Features of SLE Patients

Gender and age were recorded for all participants. All of the clinical manifestations and laboratory test findings were recorded for each of the SLE patients. The disease activity was measured by the SLE disease activity index (SLEDAI) on the same day as the MRI scan. Active disease status was defined as a SLEDAI score of higher than nine (3, 4). The depression status was evaluated by HAMD, and the anxiety status was evaluated by HAMA. Patients were diagnosed with depression when the HAMD scores were ≥ 17 and were diagnosed with anxiety when the HAMA scores ≥ 14. All participants were right-handed as assessed by the Edinburgh Handedness Inventory (5). All clinical data were collected on the MRI examination day.

### MRI Images Acquisition

The MRI images acquisition was performed by an experienced neuroradiologist. The MRI sequences were performed on all participants by using a 1.5T clinical MRI scanner manufactured by General Electric (GE) Company (Twin speed, Milwaukee, WI, USA), which was equipped with a birdcage head coil. Supportive foam pads were used to minimize the head movement. A rapid sagittal localizer scan was acquired to confirm the alignment. Normal T1 and T2 MRI scans were taken to exclude obvious structural abnormalities.

A set of conscious rs-fMRI scans was taken on each participant according to the standard protocols. Each participant was required to keep quiet and sober without active and intentional thinking. We used a gradient echo type echo planar imaging (GRE-EPI) technique with the following parameters: repetition time (TR) = 2,000 ms, echo time (TE) = 40 ms, matrix size = 64 × 64, thickness = 5 mm with an interslice gap of 1 mm, field of view = 240 mm, flip angle = 90°, number of excitation (NEX) 2.00, time point = 160. The whole-brain images were acquired in axial planes parallel to the anterior commissure-posterior commissure line. For each participant, 24 continuous slices were acquired and the total fMRI scan time was 320 s. All of the images were re-evaluated for imaging quality by the neuroradiologist.

### Data Processing

The Digital Imaging and Communications in Medicine (DICOM) image data were processed via the MRIcro software (version 1.40, Chris Rorden's Neuropsychology Laboratory, University of South Carolina, Columbia, SC, USA; http://www.mricro.com). All data were analyzed via statistical parametric mapping (SPM) 8 (Wellcome Department of Cognitive Neurology, Institute of Neurology, London, UK; http://www.fil.ion.ucl.ac.uk/) based on MATLAB 7.1 (The MathWorks, Inc. Natick, MA, USA). The images of the first 10 time points were discarded to allow the subjects to adapt to the MRI scanner and to minimize the effects of magnetic inhomogeneity. The remaining images were corrected for slice-time and head movement. Each individual image was normalized and transformed into the standardized Montreal Neurological Institute (MNI) template. Then, the images were resampled at the 3 × 3 × 3 mm scale. A filter with a full-width at half maximum (FWHM) of 10 mm was used to remove the noise for each normalized image. A bandpass filter with a range of 0.01–0.08 Hz was applied to reduce the effects of low-frequency drift and high-frequency respiratory or cardiac noises.

### Analysis of Regional Homogeneity (ReHo)

We used the Resting-State fMRI Data Analysis Toolkit (REST) (http://www.restfmri.net) to calculate the Kendall's coefficient concordance (KCC) value for each voxel. Then, we obtained the ReHo values to provide a voxel-based measurement of the brain activity. We used two-sample *t*-tests to analyze the differences in ReHo values between the SLE and HC groups with a false discovery rate (FDR) correction (*p* < 0.05, cluster > 34 voxels). We also analyzed the correlations between ReHo values and SLEDAI, HAMA and HAMD scores with a false discovery rate (FDR) correction (*p* < 0.05, cluster > 34 voxels). The differences between the demographic characteristics of the SLE and HC groups were analyzed by SPSS 20.0 (IBM Inc. Armonk, NY, USA). The results were statistically significant when *p* < 0.05. All of the statistical tests were two-sided.

## Results

### Demographic and Clinical Information

The mean age of the 118 SLE patients was 28.6 years old (standard deviation (*SD*) = 7.7). The mean disease duration of SLE patients was 19.2 months (*SD* = 20.8). The age and gender were comparable between the SLE and HC group. Detailed results are shown in Table 1.

**Table 1:** Demographic and clinical characteristics of SLE and HC groups.

	**SLE (*n* = 118)**	**HC (*n* = 81)**	***t***	***p***
Age (year, Mean ± SD)	28.6 ± 7.7	29.0 ± 7.9	−0.375	0.708
Female /Male	98/20	67/14	0.004	0.951
Disease duration (m, Mean ± SD)	19.2 ± 20.8	NA		
SLEDAI (Mean ± SD)	10.3 ± 6.8	NA		
HAMA (Mean ± SD)	7.1 ± 5.3	NA		
HAMD (Mean ± SD)	8.9 ± 5.8	NA		

*SLE, systemic lupus erythematosus; HC, healthy control; SD, standard deviation; NA, not applicable; SLEDAI, SLE disease activity index; HAMA, Hamilton Anxiety Scale; HAMD, Hamilton Depression Scale*.

### The ReHo Value Differences Between the SLE and HC Groups

In the study, four SLE patients and six HC subjects were excluded due to brain movement if the translation exceeded 2 mm or if the rotation exceeded 2. Data from the remaining 114 SLE patients and 75 HC subjects were analyzed. The average head motion assessed by the mean framewise displacement (FD)_Jenkinson index between the two groups were comparable (0.0475 ± 0.0235 vs. 0.0547 ± 0.0273, *p* = 0.066) (6).

**Figure 1:**
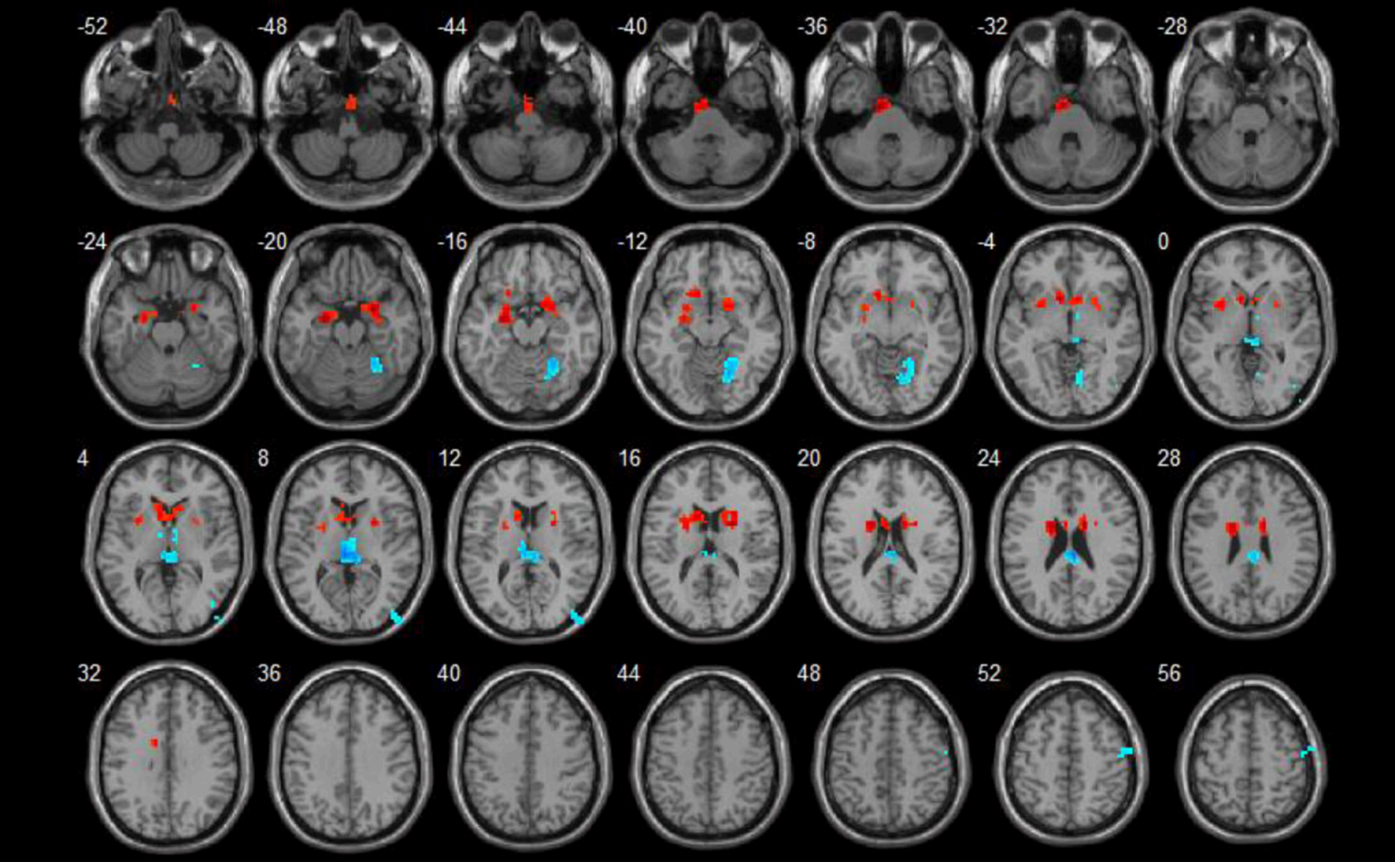
ReHo value differences between SLE and HC groups (*p* < 0.05). Red spots show areas with increased ReHo values in SLE patients, while blue ones show those with decreased ReHo values.

In the conscious resting state, the ReHo values increased in areas including the left parahippocampal gyrus and right uncus. Brain areas with decreased ReHo values included the right fusiform gyrus and left thalamus. Detailed results are shown in Table 2 and Figure 1.

**Table 2:** ReHo values in non-NPSLE patients.

**Versus HC**	**Brain area**	**Cluster size (voxel)**	**MNI coordinate (peak value)**			***T*-value (peak value)**	**Effect size**
			**X**	**Y**	**Z**		
Increased	Left parahippocampal gyrus	135	−24	−6	−18	5.97	0.41
	Right uncus	232	27	3	−21	5.44	0.48
Decreased	Right fusiform gyrus	143	21	−60	−12	−4.54	−0.34
	Left thalamus	208	−3	−27	9	−5.57	−0.43

*HC, healthy control; MNI, Montreal Neurological Institute*.

### Correlations Between the ReHo Values and Disease Activity and Depressive and Anxiety Statuses

**Figure 2:**
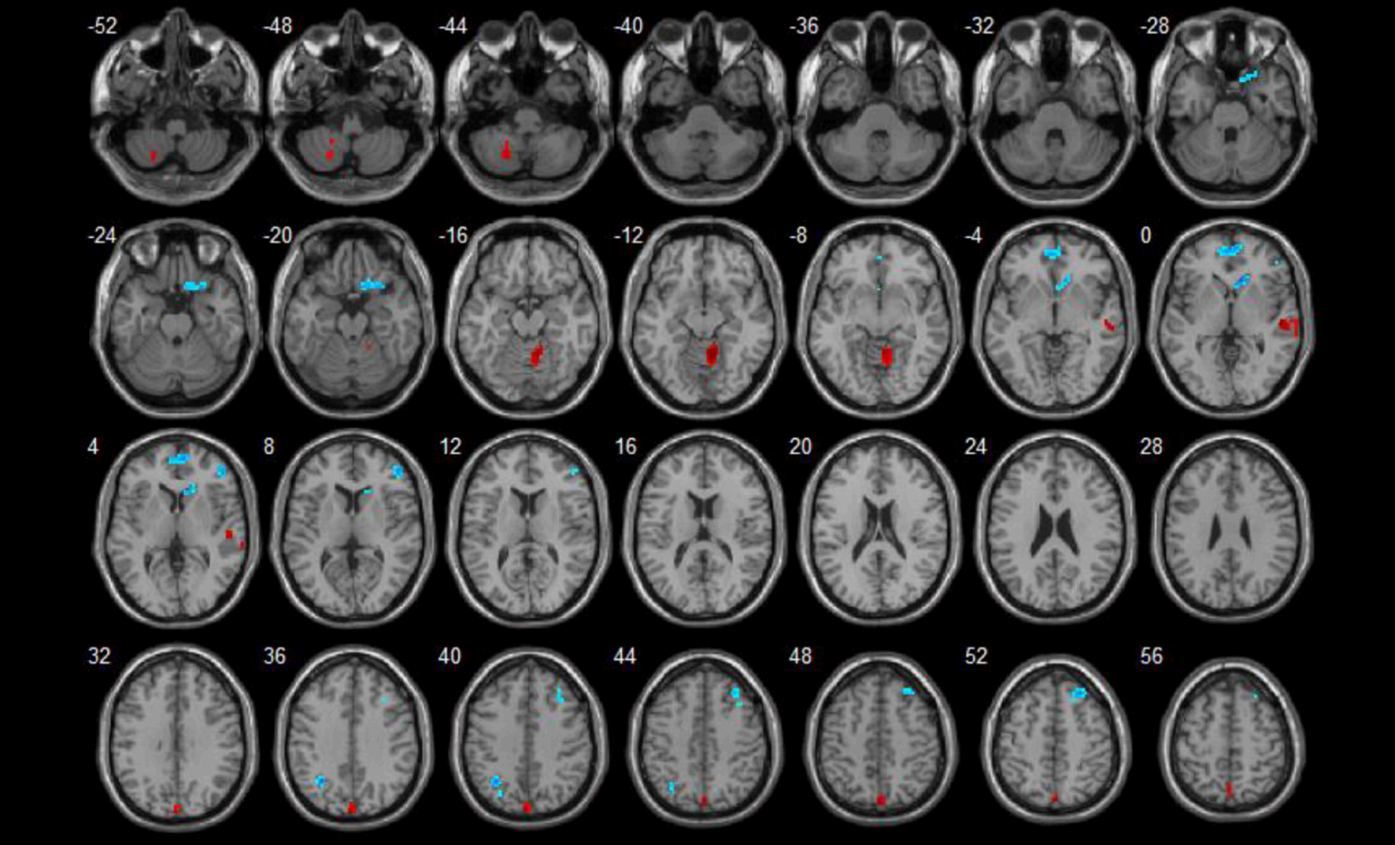
Correlations between ReHo values and disease activity (*p* < 0.05). Red spots show areas with positive correlations with SLEDAI, while blue ones show areas with negative correlations.

Brain areas that showed positive correlations with SLEDAI included: the right cerebellum anterior lobe, left cerebellum posterior lobe, and right superior temporal gyrus. Brain areas that showed negative correlations with SLEDAI were the right medial and inferior frontal gyrus. Detailed results are shown in Table 3 and Figure 2.

**Table 3:** Correlations of ReHo values between brain areas and SLEDAI.

**Correlations**	**Brain area**	**Cluster size (voxel)**	**MNI coordinate (peak value)**			**T value (peak value)**
			**X**	**Y**	**Z**	
Positive	Right cerebellum anterior lobe	57	9	−51	−12	4.08
	Left cerebellum posterior lobe	44	−24	−63	−54	3.32
	Right superior temporal gyrus	34	54	−21	0	3.92
Negative	Right medial frontal gyrus	73	6	57	0	−3.87
	Right inferior frontal gyrus	47	30	18	−21	−3.69
		34	48	45	6	−4.34

*MNI, Montreal Neurological Institute*.

**Figure 3:**
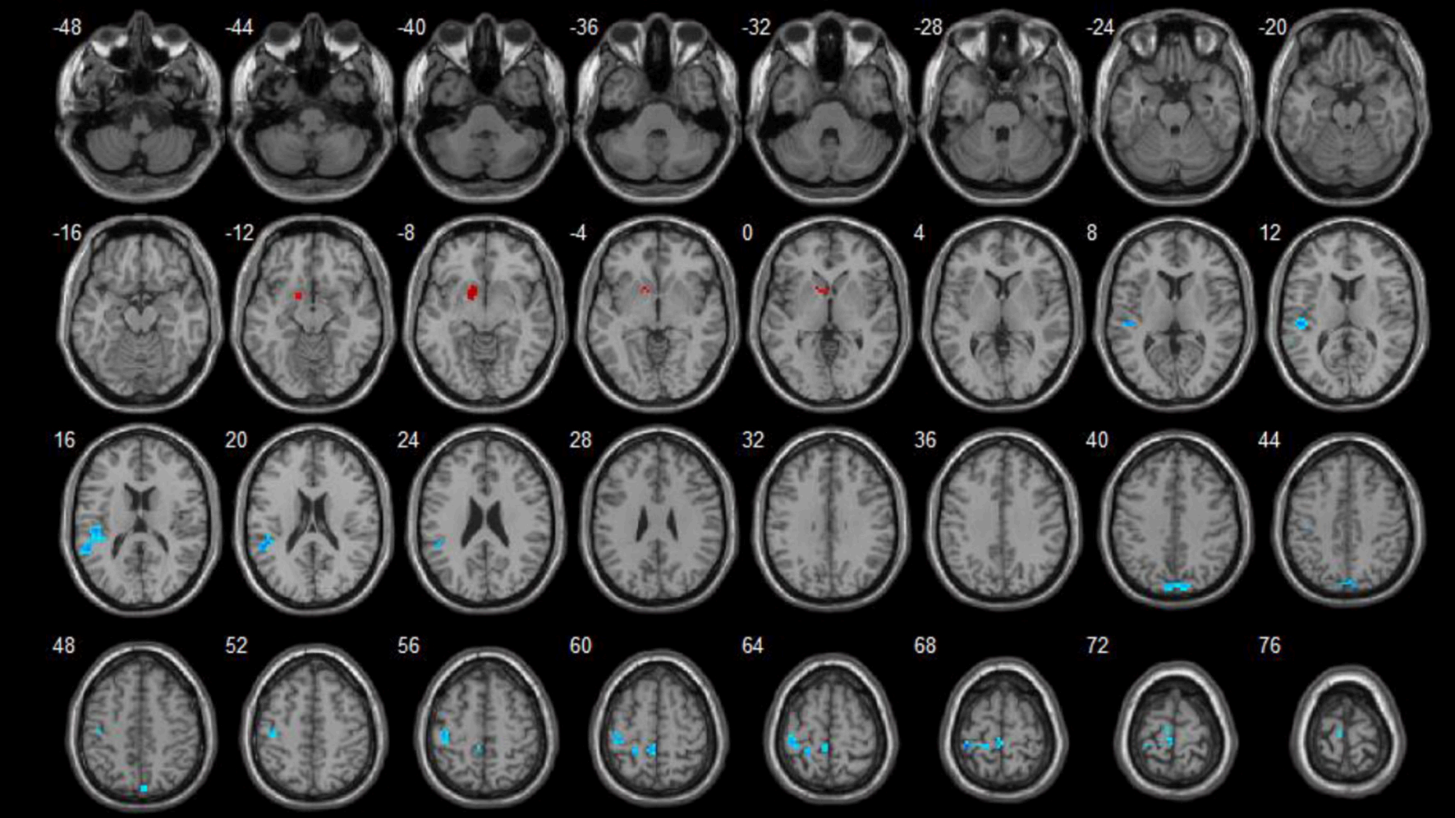
Correlations between ReHo values and HAMA scores (*p* < 0.05). Blue spots show areas with negative correlations with HAMA.

Brain areas which showed negative correlations with HAMA included: the left paracentral lobule, left postcentral gyrus, right precuneus, and left superior temporal gyrus. Detailed results are shown in Table 4 and Figure 3.

**Table 4:** Correlations of ReHo values between brain areas and HAMA.

**Correlations**	**Brain area**	**Cluster size (voxel)**	**MNI coordinate (peak value)**			***T*-value (peak value)**
			**X**	**Y**	**Z**	
Negative	Left paracentral lobule	36	−6	−39	60	−3.49
	Left postcentral gyrus	98	−36	−36	69	−4.07
	Right precuneus	59	6	−84	45	−3.53
	Left superior temporal gyrus	106	−51	−45	18	−4.10

*MNI, Montreal Neurological Institute*.

**Figure 4:**
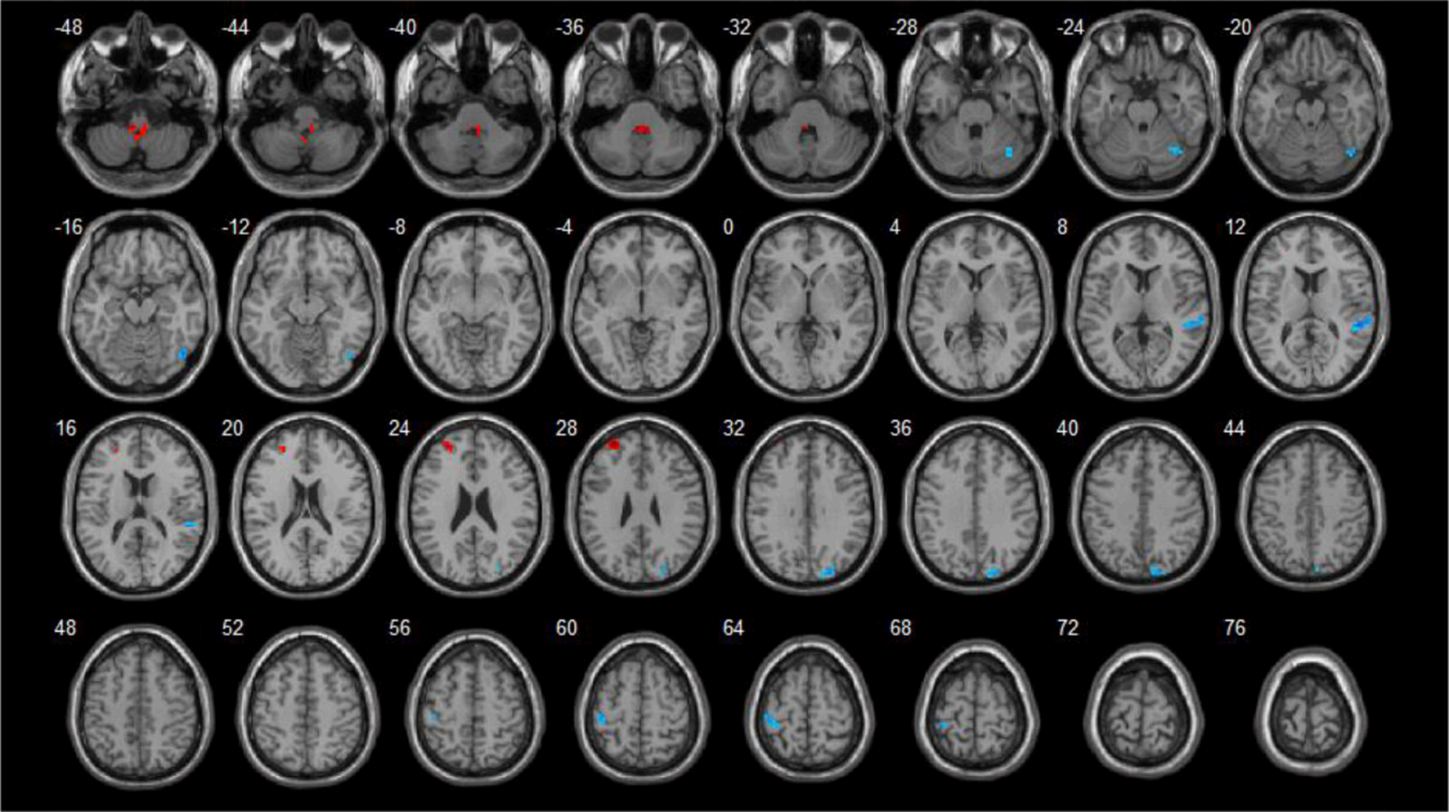
Correlations between ReHo values and HAMD scores (*p* < 0.05). Blue spots showed areas with negative correlation with HAMD.

Brain areas that showed negative correlations with HAMD included: the right cuneus, left postcentral gyrus, right superior temporal gyrus, and right fusiform gyrus. Detailed results are shown in Table 5 and Figure 4.

**Table 5:** Correlations of ReHo values between brain areas and HAMD.

**Correlations**	**Brain area**	**Cluster size (voxel)**	**MNI coordinate (peak value)**			***T*-value (peak value)**
			**X**	**Y**	**Z**	
Negative	Right cuneus	46	24	−87	33	−3.36
	Left postcentral gyrus	35	−42	−27	63	−3.40
	Right superior temporal gyrus	39	54	−36	12	−3.81
	Right fusiform gyrus	36	48	−66	−18	−3.72

*MNI, Montreal Neurological Institute*.

## Discussion

Conventional MRI and fMRI are widely used in evaluations of the brain structure and activity, respectively. Functional MRI has been used to reveal brain activities, such as working memory, depression and anxiety status, both in the normal population and in patients with several types of diseases (7, 8). As a non-invasive imaging technique, fMRI is widely used in the early diagnosis, differential diagnosis, and monitoring of neuropsychiatric diseases. Since the Ogawa team reported the blood oxygenation level dependent (BOLD) method, it has been used for the detection of regional neural activity, in task-related situations and in the conscious resting state (9). The regional homogeneity (ReHo) method is used to evaluate the regional brain synchronization by measuring the time series of a given voxel and its nearby voxels in the conscious resting state. ReHo values reflect regional brain synchronization, and increased or decreased values may represent abnormal regional brain activity (10).

In task-related fMRI studies, the participants showed high activation of target brain regions with relatively less disturbance. Task-related fMRI has been used in SLE patients to evaluate their brain activations, including motor function, working memory, and emotional activities. Rocca et al. found that NPSLE patients had more movement-associated brain activations than did the HC group in the motor function test. There were also strong correlations between the activations and the extent and severity of brain damages (11). Fitzgibbon et al. (12) found greater activations in the frontoparietal lobe of NPSLE patients than that of the HC group in working memory tasks, which might suggest brain dysfunction and the recruitment of extra brain areas, such as for compensation. Mackay et al. found that SLE patients with short disease durations showed more cortical activations in working memory tasks and fearful paradigms than did patients with long disease durations. This finding suggests that compensation could occur in a relatively short time, and long-term disease might result in severe neural damage and low activations (13). Conscious rs-fMRI could reveal the resting state brain function or brain networks. The default mode network (DMN) is a network of brain areas including the posterior cingulate cortex (PCC), the ventral anterior cingulate cortex (ACC), and other brain areas. It is the network for self-related cognitive activity and monitoring of the internal mental landscape. It is activated during the conscious resting state and is deactivated during complicated tasks (14–20). Lin et al. (21) found that non-NPSLE patients showed higher brain activations in the DMN and in the cerebellum and that the activations of the cerebellum were positively correlated with disease activities. Recently, Nystedt et al. (22) found that SLE patients with or without neuropsychiatric manifestations had brain connectivity changes in the DMN and other related brain areas, and these changes might reveal both brain damage and compensations.

Several studies have proven that in SLE patients, brain dysfunction could occur before structural changes, and the kinds of brain damage detected by different imaging methods were quite different in patients with or without neuropsychiatric symptoms (23–27). Therefore, we conducted this study to find possible brain dysfunction by conscious rs-fMRI and explored their associations with disease activity, depression and anxiety status in a relatively large sample of non-NPSLE patients.

We found that the ReHo values of non-NPSLE patients were decreased in brain areas including the fusiform gyrus and thalamus, and there was a negative correlation between the fusiform gyrus and the HAMD score. The fusiform gyrus is mainly involved in the visual recognition network. It is associated with emotional face and character processing, visual attention and learning process (28, 29). Mak et al. found that the fusiform gyrus showed decreased activities in non-NPSLE patients during the executive function test (30). A recent study showed cortical thickness reductions in the fusiform gyrus and in the lingual gyrus and abnormal resting-state functional connectivity (RSFC) in non-NPSLE patients. It suggested that cortical abnormalities might affect brain functional connectivity in non-NPSLE patients (31). These findings are consistent with our finding. The thalamus is crucial as a relay station in the brain. It can relay information between the subcortex and the cortex for most sensory systems. The increased activities of the thalamus in the resting state were reported in major depressive patients (29, 32). Nystedt et al. found hyperconnectivity of the thalamus in non-NPSLE patients, which might suggest a compensatory mechanism (22).

Increased ReHo values in the left parahippocampal gyrus and in the right uncus were found. They are both part of the limbic lobe, which also consists of the hippocampus, the cingulate gyrus, and so on. In another study, we found decreased bilateral hippocampal volume and density in non-NPSLE patients (unpublished results). In a comprehensive perspective, these results might suggest structural damage and functional compensatory mechanisms in the limbic lobe. The limbic lobe is involved in several brain activities, including motivation, emotion, learning, and the memory process. NPSLE patients with greater hippocampus activity and hippocampal functional connectivity were found to have better learning efficiency (33). It was reported that SLE patients without cognitive impairments had increased activity in the hippocampus, and the compensation of the brain function was decreased as the disease eventually progressed (34). As we mentioned earlier, Rocca et al. found that there were positive correlations between the activations and brain damage in NPSLE patients (11). This finding might suggest that there was a compensatory mechanism during the early stage of SLE, and the brain function was eventually damaged during the advanced stage (22).

Our study showed a positive correlation between disease activity and the cerebellum, which might suggest a compensatory mechanism. Ren et al. found that the activity of the cortico-basal ganglia-thalamic-cortical circuit and amygdala-hippocampus coupling was decreased, while an increased cerebellar-frontal activity could compensate for the brain function in cognitive tasks in non-NPSLE patients (35). Lin et al. also found a positive correlation between the cerebellar activity and the SLEDAI score during the resting stage in non-NPSLE patients. They considered that the cerebellum could be part of the executive control network and related to the pathogenesis of NPSLE (21).

We found that the right medial and inferior frontal gyrus were both negatively correlated with the SLEDAI score. The frontal gyrus is the center of emotional regulation and voluntary movement. Fitzgibbon et al. found fronto-parietal cortex hyperactivation and positive correlations with disease activities in NPSLE patients in working memory tasks (12), whereas Hou et al. found similar results in non-NPSLE patients during the resting state (36). Rocca et al. found activations in the frontal and parietal lobes in NPSLE patients during motor tasks. There were positive correlations between the activation of sensorimotor areas and the extent and severity of brain damage (11). We failed to find this compensation effect and found quite opposite results. One possible explanation is that our participants had a relatively short disease duration and we had a larger sample. The frontal gyrus may play a complicated role in SLE patients, and more studies are needed to elucidate the exact role.

The HAMA and HAMD scores were negatively correlated with similar brain areas, including the left paracentral lobule and postcentral gyrus, the right precuneus and cuneus, and the bilateral superior temporal gyrus. The postcentral gyrus is the primary somatosensory cortex, and the paracentral lobule controls motor and sensory innervations. The precuneus is involved in the episodic memory, visuospatial processing, and self-consciousness. The cuneus is involved in basic visual processing. The superior temporal gyrus contains the primary auditory cortex and Wernicke's area, which is involved in the comprehension of language. The abnormal brain activities in the precuneus, cuneus and superior temporal gyrus of SLE patients were reported in previous studies (11, 21, 30, 37). However, the correlations between depression and the anxiety status within these areas in SLE patients were not reported previously. More studies are needed to confirm our finding and to explore the underlying mechanism.

There are still some flaws in our study. One is that we only used ReHo values to reflect the brain activity, because there were other available methods, such as the amplitude of low frequency fluctuations (ALFF). Zou et al. found that the reliability of ReHo values was good with standard protocols including nuisance correction, enough scan durations, and fast sampling rates (38). In our study, we considered the ReHo value to be feasible for reflecting the brain activity.

We found that the brain areas with decreased ReHo values were in the fusiform gyrus and thalamus, while the areas with increased ReHo values were in the limbic lobe. We found that SLEDAI showed a positive correlation with brain areas in the cerebellum and a negative correlation with brain areas in the frontal gyrus. Several brain areas showed negative correlations with depression and anxiety statuses. These results gave us some clues on how the brain synchronization changes in non-NPSLE patients. Early detection and proper treatment of brain dysfunction might prevent progression to NPSLE. More studies are needed to reveal the underlying mechanisms.

## Ethics Statement

This study was carried out in accordance with the recommendations of the clinical trial guidelines of the Institutional Review Board of Kunming Medical University with written informed consent from all subjects. All subjects gave written informed consent in accordance with the Declaration of Helsinki. The protocol was approved by the Institutional Review Board of Kunming Medical University.

## Author Contributions

SL and YC were responsible for the management of the research and writing the article. ZX, AL, ZL, and YZ were responsible for recruiting and following up the patients. CL and HY were responsible for doing MRI. XX, BS, and LX were responsible for the consultation of the research. JX was responsible for the whole research and article.

### Conflict of Interest Statement

The authors declare that the research was conducted in the absence of any commercial or financial relationships that could be construed as a potential conflict of interest.
